# Cleavage Intercondylar Fracture of the Femur: A Case Report

**DOI:** 10.7759/cureus.25127

**Published:** 2022-05-18

**Authors:** Firas Chaouch, Saber Rabhi, Youssef Othman, Makram Zrig, Abderrazek Abid

**Affiliations:** 1 Orthopaedics and Traumatology, Monastir University School of Medicine, Fattouma Bourguiba Hospital, Monastir, TUN

**Keywords:** osteoarthritis, cleavage, distal femur, fracture, intercondylar

## Abstract

Distal femur fractures account for less than 1% of all fractures and about 3 to 6% of all femoral fractures. Several classifications have been described but some types of distal femur fractures escape them such as the cleavage intercondylar fracture of the femur. To our knowledge, there have been only four cases reported in the literature. The authors report a case of a 32-year-old woman who presented at the emergency department with a cleavage intercondylar fracture of the left femur. The patient was treated with a long leg cast for 6 weeks, followed by physiotherapy and full weight-bearing. After 4 months, the evolution was favorable: the patient was asymptomatic and regained full knee range of motion. After 10 years of follow-up, there was no clinical or radiological evidence of knee osteoarthritis.

## Introduction

Distal femur fractures account for less than 1% of all fractures and about 3 to 6% of all femoral fractures [1]. These fractures can be caused by either high-energy trauma or low-energy trauma. High-energy trauma including road traffic accidents and sports accidents are more likely to occur in men aged between 15 and 50, whereas low-energy trauma such as falls from standing height are more likely to lead to distal femur fractures in women aged 50 and above. In both cases, axial loading of the leg is the most common mechanism of injury [2]. Because of the inherent complexity of these injuries, several classifications have been described to allow adequate documentation, plan treatment, and evaluate the results. However, some types of distal femur fractures escape them. The authors report a rare case of cleavage intercondylar fracture of the femur through which they try to specify the epidemiological features, the mechanism of injury, the pathogenesis, therapeutic attitudes, and the long-term prognosis of this type of fracture pattern.

## Case presentation

**Figure 1:**
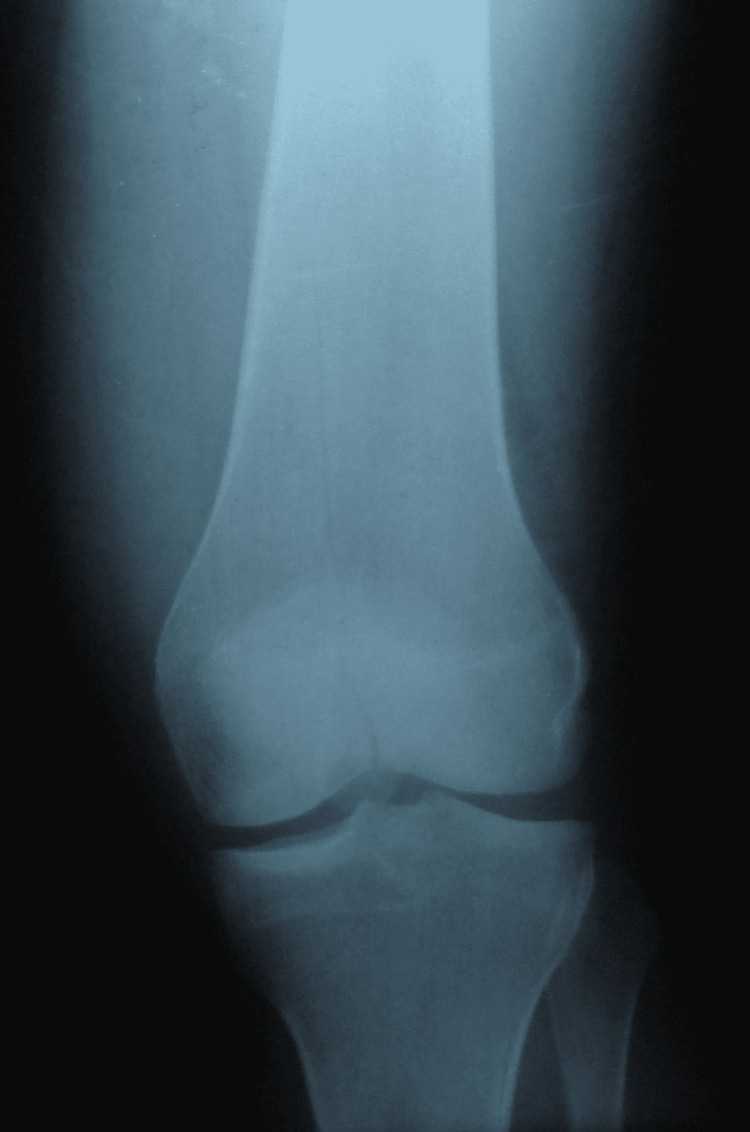
Plain radiograph showing a vertical fracture line of the femoral metaphysis extending to the intercondylar notch without separation of a bone fragment

A 32-year-old woman was a victim of a blow with a stick on the anterior aspect of her left knee while it was flexed to 90°. She described severe knee pain and was unable to weight-bear. The clinical examination found an effusion of the left knee and pain on the palpation of the femoral condyles. Mobilization of the knee was very painful. Plain radiographs showed a vertical fracture line of the femoral metaphysis extending to the intercondylar notch without separation of a bone fragment (Figure 1).

**Figure 2:**
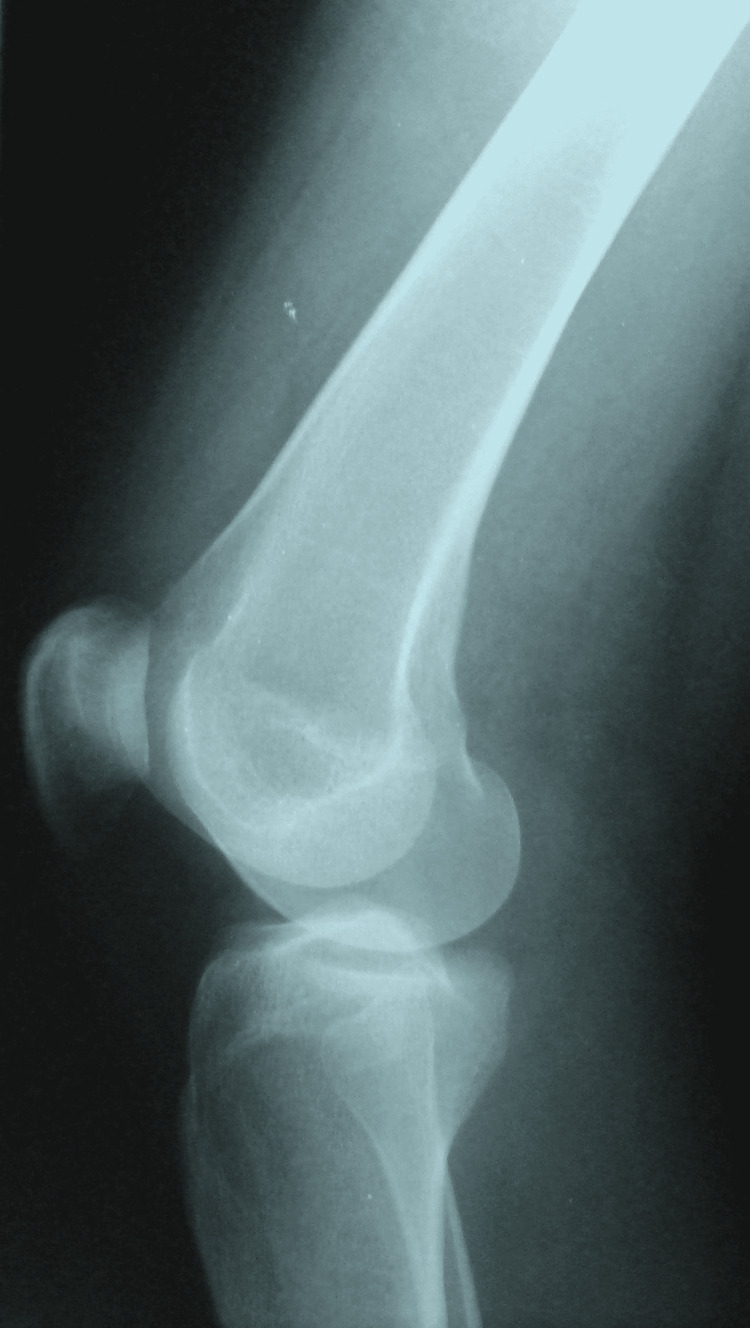
Lateral view did not show signs of fracture

On lateral view (Figure 2), the fracture was not visible.

**Figure 3:**
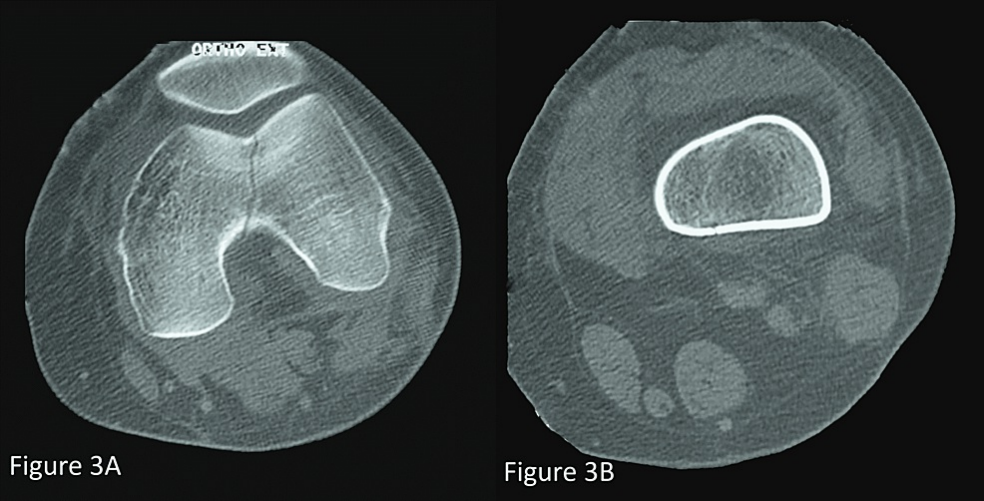
CT scan showing an intercondylar fracture without cortical damage or displacement

A computed tomography (CT) scan of the left knee (Figure 3) was performed and showed an intercondylar fracture without cortical damage or displacement. This fracture was associated with minimal hyperdensity of the subchondral bone of proximal tibial and the two femoral condyles reflecting bone edema.

Since the fracture was not displaced, we opted for nonsurgical management with a long leg cast for 6 weeks, followed by physiotherapy and full weight-bearing. After 4 months, the evolution was favorable: the patient was asymptomatic and regained full range of motion. After 10 years of follow-up, there was no clinical or radiological evidence of knee osteoarthritis.

## Discussion

Fractures of the lower end of the femur are relatively rare, accounting for less than 1% of all fractures and 6% of all femoral fractures [1]. Few classifications have been proposed to allow adequate documentation, plan treatment, and evaluate the results. Neer et al. described early in 1967 one of the simpler classification systems [3]. In 1980, Seinsheimer published his system where he classified the fractures of the distal femur into four types [4]. Actually, the most commonly used classification for distal femur fractures is the AO/Orthopedic Trauma Association (OTA) system described by Müller and his colleagues [5]. The purpose of these systems is to analyze and categorize the variety of these fractures. Although, some fractures cannot be included in any classification, such as cleavage intercondylar fractures of the femur. As far as the authors’ knowledge, there have been only four cases reported in the literature [6,7].

The mechanism of these fractures remains unclear. Pogrund et al. reported three cases of “cleavage intercondylar fracture of the femur” and stated that this injury is caused by force transmitted through the patella [6]. Our case reinforces the theory that these fractures are caused by a direct high-energy shock on a knee flexed between 60 and 90°. In this position, the patella is located in the axis of the femur and transmits the shock wave toward the trochlea, resulting in a fracture associated with a cartilage contusion.

Anteroposterior knee radiograph shows a sagittal, vertical fracture line originating in the intercondylar notch, extending upwards into the metaphysis, and ending in the diaphysis without breaking the cortices. The CT scan confirms the continuity of the femoral cortices and detects possible patellofemoral cartilage damage.

Literature review suggests that surgical fixation for distal femur fractures has consistently proved better outcomes than the nonsurgical methods, including improved alignment, union rates, knee motion, and functional outcome. Nonsurgical management of intra-articular distal femur fractures is reserved for stable, minimally displaced fractures [8]. Furthermore, experimental studies revealed that an incongruity <2 mm can be tolerated as long as ligamentous structures are intact [9].

Stability in this fracture pattern is maintained by the integrity of the medial and lateral columns. Furthermore, in this type of fracture, there is no displacement and thus no incongruity of surface which indicates the nonoperative treatment. Management consists of restricted weight-bearing in splinting or casting and maintained for 4 to 6 weeks [10]. Radiographs should be obtained throughout the recovery process to ensure appropriate healing and monitor for displacement. After cast removal, weight-bearing should be protected until there is radiographic evidence of the union.

In our case, the short-term evolution was favorable, with consolidation and recovery of a full range of motion. In the long term, there was no clinical or radiological evidence of patellofemoral osteoarthritis. However, this complication occurred in the three cases reported in Pogrund et al.’s study, which highlights the severe concomitant damage to the articular cartilage of the patella and intercondylar region and indicates a long-term follow-up [6].

## Conclusions

Cleavage intercondylar fracture of the distal femur is a rare injury, especially at this age and with no background of illness. This type of fracture does not exist in the classification of distal femur fractures. In this case, the authors opted for nonoperative treatment, but long-term follow-up is recommended to recognize possible patellofemoral osteoarthritis.
